# Epidemiology of childhood acute leukemias in marginalized populations of the central-south region of Mexico: results from a population-based registry

**DOI:** 10.3389/fonc.2024.1304263

**Published:** 2024-02-20

**Authors:** Janet Flores-Lujano, Aldo Allende-López, David Aldebarán Duarte-Rodríguez, Erika Alarcón-Ruiz, Lizbeth López-Carrillo, Teresa Shamah-Levy, Mariano E. Cebrián, Ma. del Rocío Baños-Lara, Diana Casique-Aguirre, Jesús Elizarrarás-Rivas, Javier Antonio López-Aquino, Miguel Ángel Garrido-Hernández, Daniela Olvera-Caraza, Vanessa Terán-Cerqueda, Karina Beatriz Martínez-José, Pierre Mitchel Aristil-Chery, Enoch Alvarez-Rodríguez, Wilfrido Herrera-Olivares, Guillermo J. Ruíz-Arguelles, Lénica Anahí Chavez-Aguilar, Aquilino Márquez-Toledo, Lena Sarahi Cano-Cuapio, Nuria Citlalli Luna-Silva, Maria Angélica Martínez-Martell, Anabel Beatriz Ramirez-Ramirez, Laura Elizabeth Merino-Pasaye, César Alejandro Galván-Díaz, Aurora Medina-Sanson, Maria de Lourdes Gutiérrez-Rivera, Jorge Alfonso Martín-Trejo, Emmanuel Rodriguez-Cedeño, Vilma Carolina Bekker-Méndez, María de los Ángeles Romero-Tlalolini, Astin Cruz-Maza, Gerardo Juárez-Avendaño, Sonia Mayra Pérez-Tapia, Juan Carlos Rodríguez-Espinosa, Miriam Carmina Suárez-Aguirre, Fernando Herrera-Quezada, Anahí Hernández-Díaz, Lizbeth Alondra Galván-González, Minerva Mata-Rocha, Amanda Idaric Olivares-Sosa, Haydeé Rosas-Vargas, Silvia Jiménez-Morales, Mariana Cárdenas-González, María Elena Álvarez-Buylla Roces, Célida Duque-Molina, Rosana Pelayo, Juan Manuel Mejía-Aranguré, Juan Carlos Núñez-Enriquez

**Affiliations:** ^1^ Unidad de Investigación Médica en Epidemiología Clínica, Unidad Médica de Alta Especialidad (UMAE) Hospital de Pediatría “Dr. Silvestre Frenk Freund”, Centro Médico Nacional Siglo XXI, Instituto Mexicano del Seguro Social (IMSS), Mexico City, Mexico; ^2^ División de Desarrollo de la Investigación, Coordinación de Investigación en Salud, Instituto Mexicano del Seguro Social (IMSS), Mexico City, Mexico; ^3^ División de Estudios de Posgrado e Investigación, Tecnológico Nacional de México, Instituto Tecnológico de Ciudad de Madero, Ciudad Madero, Tamaulipas, Mexico; ^4^ Centro de Investigación en Salud Poblacional, Instituto Nacional de Salud Pública (INSP), Cuernavaca, Morelos, Mexico; ^5^ Centro de Investigación en Evaluación y Encuestas, Instituto Nacional de Salud Pública (INSP), Cuernavaca, Morelos, Mexico; ^6^ Departamento de Toxicología, Centro de Investigación y de Estudios Avanzados (CINVESTAV), Instituto Politécnico Nacional, Mexico City, Mexico; ^7^ Facultad de Medicina, Universidad Popular Autónoma del Estado de Puebla, Puebla, Mexico; ^8^ Centro de Investigación Oncológica Una Nueva Esperanza, Universidad Popular Autónoma del Estado de Puebla, Puebla, Mexico; ^9^ Laboratorio de Citómica del Cáncer Infantil, Centro de Investigación Biomédica de Oriente, Instituto Mexicano del Seguro Social, Delegación Puebla, Puebla, Mexico; ^10^ Consejo Nacional de Humanidades, Ciencias y Tecnologías (CONAHCYT), Mexico City, Mexico; ^11^ Coordinación de Investigación en Salud, Instituto Mexicano del Seguro Social, Oaxaca, Mexico; ^12^ Coordinación Clínica de Educación e Investigación en Salud de la UMF No. 1, Instituto Mexicano del Seguro Social, Oaxaca, Mexico; ^13^ Servicio de Oncohematología Pediátrica, Hospital para el Niño Poblano, Secretaria de Salud (SS), Puebla, Mexico; ^14^ Servicio de Oncohematología Pediátrica, Instituto Mexicano del Seguro (IMSS) Unidad Médica de Alta Especialidad (UMAE) Centro Médico Nacional (CMN) Hospital de Especialidades Dr. Manuel Ávila Camacho, Puebla, Mexico; ^15^ Servicio de Hematología, Instituto Mexicano del Seguro (IMSS) Unidad Médica de Alta Especialidad (UMAE) Centro Médico Nacional (CMN) Hospital de Especialidades Dr. Manuel Ávila Camacho, Puebla, Mexico; ^16^ Departamento de Enseñanza e Investigación, Instituto de Seguridad y Servicios Sociales de los Trabajadores al Servicio de los Poderes del Estado de Puebla (ISSSTEP), Puebla, Mexico; ^17^ Servicio de Oncohematología Pediátrica, Instituto de Seguridad y Servicios Sociales de los Trabajadores al Servicio de los Poderes del Estado de Puebla (ISSSTEP), Puebla, Mexico; ^18^ Servicio de Hematología Pediátrica, Instituto de Seguridad y Servicios Sociales de los Trabajadores del Estado (ISSSTE), Puebla, Mexico; ^19^ Servicio de Oncohematología, Hospital General del Sur Dr. Eduardo Vázquez Navarro, Secretaria de Salud (SS), Puebla, Mexico; ^20^ Centro de Hematología y Medicina Interna, Clínica Ruiz, Puebla, Mexico; ^21^ Departamento de Pediatría, Hospital Universitarios Puebla (BUAP), Puebla, Mexico; ^22^ Servicio de Oncología Pediátrica, Hospital Infantil de Tlaxcala, Secretaria de Salud (SS), Tlaxcala, Mexico; ^23^ Servicio de Hemato-Oncología Pediátrica, Hospital de la Niñez Oaxaqueña “Dr. Guillermo Zárate Mijangos”, Secretaria de Salud y Servicios de Salud Oaxaca (SSO), Oaxaca, Mexico; ^24^ Servicio de Oncocrean, Hospital General de Zona 01 “Dr. Demetrio Mayoral Pardo” Instituto Mexicano del Seguro Social (IMSS), Oaxaca, Mexico; ^25^ Servicio de Hematología Pediátrica, Centro Médico Nacional 20 de Noviembre, Instituto de Seguridad y Servicios Sociales de los Trabajadores del Estado (ISSSTE), Mexico City, Mexico; ^26^ Departamento de Oncología Pediátrica, Instituto Nacional de Pediatría, Secretaría de Salud, Mexico City, Mexico; ^27^ Departamento de Hemato-Oncología, Hospital Infantil de México Federico Gómez, Secretaría de Salud, Mexico City, Mexico; ^28^ Servicio de Oncología, Unidad Médica de Alta Especialidad (UMAE) Hospital de Pediatría “Dr. Silvestre Frenk Freund”, Centro Médico Nacional Siglo XXI, Instituto Mexicano del Seguro Social (IMSS), Mexico City, Mexico; ^29^ Servicio de Hematología, Unidad Médica de Alta Especialidad (UMAE) Hospital de Pediatría “Dr. Silvestre Frenk Freund”, Centro Médico Nacional Siglo XXI, Instituto Mexicano del Seguro Social (IMSS), Mexico City, Mexico; ^30^ Servicio de Hematología, Unidad Médica de Alta Especialidad, Hospital General “Dr. Gaudencio González Garza”, Centro Médico Nacional La Raza, Instituto Mexicano del Seguro Social (IMSS), Mexico City, Mexico; ^31^ Unidad de Investigación Biomédica en Inmunología e Infectología, Hospital de Infectología “Dr. Daniel Méndez Hernández”, “La Raza”, Instituto Mexicano del Seguro Social (IMSS), Mexico City, Mexico; ^32^ Laboratorio de Biología Molecular, Facultad de Medicina y Cirugía, Universidad Autónoma Benito Juárez de Oaxaca (UABJO), Oaxaca, Mexico; ^33^ Unidad de Desarrollo e Investigación en Bioterapéuticos (UDIBI), Escuela Nacional de Ciencias Biológicas, Instituto Politécnico Nacional, Mexico City, Mexico; ^34^ Laboratorios Juárez Oaxaca, Oaxaca, Mexico; ^35^ Facultad de Biotecnología, Universidad Popular Autónoma del Estado de Puebla, Puebla, Mexico; ^36^ Unidad de Investigación Médica en Genética Humana, Unidad Médica de Alta Especialidad (UMAE) Hospital de Pediatría “Dr. Silvestre Frenk Freund”, Centro Médico Nacional Siglo XXI, Instituto Mexicano del Seguro Social (IMSS), Mexico City, Mexico; ^37^ Dirección de Educación e Investigación, Unidad Médica de Alta Especialidad (UMAE) Hospital de Pediatría “Dr. Silvestre Frenk Freund”, Centro Médico Nacional Siglo XXI, Instituto Mexicano del Seguro Social (IMSS), Mexico City, Mexico; ^38^ Laboratorio de Innovación y Medicina de Precisión, Núcleo A. Instituto Nacional de Medicina Genómica (INMEGEN), Mexico City, Mexico; ^39^ Dirección de Prestaciones Médicas, Instituto Mexicano del Seguro Social (IMSS), Mexico City, Mexico; ^40^ Unidad de Oncoinmunología y Citómica, Centro de Investigación Biomédica de Oriente (CIBIOR), Instituto Mexicano del Seguro Social (IMSS), Puebla, Mexico; ^41^ Unidad de Educación e Investigación en Salud, Instituto Mexicano del Seguro Social, Mexico City, Mexico; ^42^ Laboratorio de Genómica del Cáncer, Instituto Nacional de Medicina Genómica (INMEGEN), Mexico City, Mexico; ^43^ Facultad de Medicina, Universidad Nacional Autónoma de México (UNAM), México City, Mexico

**Keywords:** incidence, child, lymphoid leukemia, myeloid leukemia, registries, epidemiology, Latino

## Abstract

**Introduction:**

Acute leukemias (AL) are the main types of cancer in children worldwide. In Mexico, they represent one of the main causes of death in children under 20 years of age. Most of the studies on the incidence of AL in Mexico have been developed in the urban context of Greater Mexico City and no previous studies have been conducted in the central-south of the country through a population-based study. The aim of the present work was to identify the general and specific incidence rates of pediatric AL in three states of the south-central region of Mexico considered as some of the marginalized populations of Mexico (Puebla, Tlaxcala, and Oaxaca).

**Methods:**

A population-based study was conducted. Children aged less than 20 years, resident in these states, and newly diagnosed with AL in public/private hospitals during the period 2021-2022 were identified. Crude incidence rates (cIR), standardized incidence rates (ASIRw), and incidence rates by state subregions (ASIRsr) were calculated. Rates were calculated using the direct and indirect method and reported per million children under 20 years of age. In addition, specific rates were calculated by age group, sex, leukemia subtype, and immunophenotype.

**Results:**

A total of 388 cases with AL were registered. In the three states, the ASIRw for AL was 51.5 cases per million (0-14 years); in Puebla, it was 53.2, Tlaxcala 54.7, and Oaxaca de 47.7. In the age group between 0-19 years, the ASIRw were 44.3, 46.4, 48.2, and 49.6, in Puebla, Tlaxcala, and Oaxaca, respectively. B-cell acute lymphoblastic leukemia was the most common subtype across the three states.

**Conclusion:**

The incidence of childhood AL in the central-south region of Mexico is within the range of rates reported in other populations of Latin American origin. Two incidence peaks were identified for lymphoblastic and myeloid leukemias. In addition, differences in the incidence of the disease were observed among state subregions which could be attributed to social factors linked to the ethnic origin of the inhabitants. Nonetheless, this hypothesis requires further investigation.

## 1 Introduction

Acute leukemias (AL) are the main types of cancer in children worldwide. In Latin American countries (LAC) high incidence and mortality rates have been reported (1–5). Moreover, for some LAC an increase in leukemia mortality has been predicted by 2030 (6). Mexico is among these populations along with Argentina, Brazil, Chile, Ecuador, Guatemala, Peru, Puerto Rico, and Uruguay. According to data from the National Institute of Statistics, Geography, and Informatics of Mexico (INEGI) in 2019 (the year previous to the COVID-19 pandemic), childhood leukemias ranked first among the leading causes of death in the 5 to 9 years age group, and second in the 10 to 14 years age group, only surpassed by motor vehicle accidents (7). Consequently, the problem of childhood leukemia in Mexico such as another LAC requires effective actions aimed at prevention in order to reduce the incidence and mortality of AL.

To date, few risk factors have been found to be associated with childhood leukemia and explain less than 10% of the causes of the disease (8). One of the regions of Mexico which has been explored further in previous studies is Greater Mexico City (GMC) where it has been reported that the incidence of AL is among the highest in the world (9). Moreover, the relapses and deaths in the early phases of treatment (first year after diagnosis) are at least three times more frequent than in high-income countries despite the use of the same chemotherapy regimens (10, 11). Then, it is evident that each country and regions within countries have their own particularities for considering the study of factors associated with both the etiology and the prognosis of the disease such as the environmental and socioeconomic conditions, lifestyle, and access to health services among others (12–15).

Since January 2021 a population-based leukemia registry was implemented in the States of Puebla, Tlaxcala, and Oaxaca located in the central-south region of Mexico as a first step for investigating for the first time the incidence and mortality rates of AL in the pediatric population, the needs for hospital services, the efficacy of current treatments, the discovering of the factors associated with therapeutic failure and the environmental risks factors associated with the development of the disease.

In the present report, we display the results of AL incidence after two years of the leukemia registry implementation in Puebla, Tlaxcala, and Oaxaca.

## 2 Materials and methods

Cases with AL were recruited from public and private hospitals of Puebla, Tlaxcala, and Oaxaca from 1 January 2021 to 31 December 2022. Additionally, those patients from these Mexican States who were diagnosed and/or treated in public hospitals of GMC during the study period were registered. In this regard, it is important to highlight that the public hospitals of GMC diagnos and treat patients from almost all the States of the country because of the infrastructure of the Institutions and relatively easy access for some families to reach them. Therefore, as part of the present study, all public hospitals of GMC that in the last 10 years had attended children from Puebla, Tlaxcala, and Oaxaca also participated (See the list of the participating institutions in **Table 1**).

**Table 1:** Participating Institutions in the population-based registry of incident cases with childhood acute leukemias in Puebla, Tlaxcala and Oaxaca.

State	Hospital name/Laboratory name	Healthcare institution
**Oaxaca**	Hospital Especializado de la Niñez Oaxaqueña	SS/INSABI
	Hospital General de Zona 1 “Dr. Demetrio Mayoral Pardo”	IMSS
	Laboratorios Juárez	Private
**Puebla**	Hospital para el Niño Poblano	SS/INSABI
	UMAE Hospital de especialidades CMN Manuel Ávila Camacho (San José)	IMSS
	Hospital Regional de Alta Especialidad	ISSSTE
	Instituto de Seguridad y Servicios Sociales de los Trabajadores al Servicio de Los Poderes del Estado de Puebla	ISSSTEP
	Hospital General del Sur	INSABI
	Hospital Universitario de Puebla	BUAP/Private
	Centro de Hematología y Medicina Interna, Clínica Ruiz	Private
	Laboratorio de Oncoinmunología and Citómica del Cáncer Infantil	CIBIOR
	Centro de Investigación Oncológica Una Nueva Esperanza-UPAEP	UNE-UPAEP
**Tlaxcala**	Hospital Infantil de Tlaxcala	SS/INSABI
**Mexico City**	UMAE Hospital de Pediatría “Dr. Silvestre Frenk Freund” CMN Siglo XXI	IMSS
	Centro Médico Nacional 20 de Noviembre	ISSSTE
	Instituto Nacional de Pediatría	SS/INSABI
	Hospital Infantil de México Federico Gómez	SS/INSABI
	Hospital Juárez de México	SS/INSABI

IMSS, Instituto Mexicano del Seguro Social; SS, Secretaría de Salud; INSABI, Instituto de Salud para el Bienestar; CLUES, Clave Única de Establecimientos de Salud; UMAE, Unidad Médica de Alta Especialidad; CMN, Centro Médico Nacional; ISSSTE, Instituto de Seguridad y Servicios Sociales de los Trabajadores del Estado; A.C, Asociación Civil; UPAEP, Universidad Popular Autónoma del Estado de Puebla.

### 2.1 Ethical considerations

The protocol was approved by the National Scientific Research and Ethics Committee of the Mexican Institute of Social Security with the number R-2020-785-022. In addition, authorization was received from the Local Ethics and Research Committees of each participating institution.

### 2.2 Validity of methods

Trained and standardized personnel were assigned to each participating hospital for case registration, ensuring the identification and follow-up of incident cases of AL. Clinical and demographic data including: sex, age at diagnosis, municipality of residence, socioeconomic level (SES), and immunophenotype were recorded. In addition, face-to-face interviews were conducted with parents and tutors of patients with a confirmed diagnosis of AL in order to validate the information related to the place of residence of the families. Written informed consent was obtained from parents and informed assent was obtained from patients ≥8 years old, when feasible, to be included in the study. In this study, all parents/guardians agreed to participate in the enrollment phase. Therefore, our participation rate was 100%.

The diagnosis was established by pediatric hematologists/oncologists according to clinical features: cell morphology, immunophenotype, and genetics, as defined by the 2008 World Health Organization (WHO) classification of lymphoid neoplasms. According to ICD-O-3 and ICCC-3, the subtype of AL was classified into two groups: a) acute lymphoblastic leukemia (ALL) (9820, 9823, 9826,9827, 9831, 9833-9837, 9940, 9948); and b) acute myeloid leukemia (AML) (9840, 9860, 9861, 9866, 9867, 9870-9874, 9891, 9895-9897, 9910, 9920, 9931) (16, 17). In regard to the immunophenotype, ALL patients were classified into B-cell, T-cell, or acute leukemia of ambiguous lineage (ALAL) according to the WHO.

### 2.3 Participating institutions

#### 2.3.1 Populations and methods for incidence rates estimations

The states of Puebla, Tlaxcala, and Oaxaca were studied. Population information for these three states was obtained from the 2015-2050 censuses of the National Population Council (CONAPO). The total average annual population <20 years of age (reference population) from Puebla, Tlaxcala, and Oaxaca was 6,092,475.

Incidence rates were calculated and reported per million population under 20 years of age at the state and sub-regional levels.

### 2.4 Crude incidence rates

The state-level crude incidence rate (cIR) was calculated by dividing the number of incident cases of acute leukemia during the period 2021-2022 by the population under 20 years of age projected by the CONAPO for the years 2021-2022 (18).

This rate was calculated to estimate the global incidence and by subgroups according to: a) demographic variables: sex and age groups (0-4 years, 5-9 years, 10-14 years, 15-19 years, 0-14 years, 0-19 years); b) clinical variables: leukemia subtype (ALL, AML, OL) and immunophenotype (B-cell, T-cell, and ALAL); c) geographic unit of analysis: states (Puebla, Tlaxcala, and Oaxaca); and subregions of the three states, Puebla (Sierra Norte, Sierra Nororiental, Valle de Serdán, Angelópolis, Valle de Atlixco y Matamoros, Mixteca, Tehuacán y Sierra Negra); Tlaxcala (Norte, Oriente, Poniente, Centronorte, Centrosur, Sur); Oaxaca (Cañada, Costa, Istmo, Mixteca Oaxaqueña, Cuenca del Papaloapan, Sierra Norte; Sierra Sur, Valles Centrales de Oaxaca). (19). In addition, the cIR by subregions was calculated using the summation of the population under 20 years of age from the municipalities conforming to each subregion projected by CONAPO for the period 2016-2050 (20).

It is worth mentioning that Puebla, Tlaxcala, and Oaxaca, are comprised of 847 municipalities ranging from 23 to 517,395 inhabitants under 20 years of age per municipality. Therefore, calculating the rates at the municipal level would generate unstable rates, and consequently random fluctuations, and this observation would not be accurate for this disease. For this reason, it was decided to calculate incidence rates by state subregions as smaller geographic units of analysis with the intention of identifying areas with high incidence rates of acute leukemias and reducing this difficulty derived from the scarcity of data at the municipal level (numerous strata with insufficient or no data). (New York State 1999; Washington State Department of Health 2012).

### 2.5 Standardized incidence rates

Standardized incidence rates (sIR) were calculated using both direct and indirect methods, which were also reported per million children under 20 years of age. The direct method compared age-standardized incidence rates of AL worldwide (ASIRw), with the reference being the world population reported by the WHO for the period 2000-2025 (21). The indirect method was used to calculate standardized incidence rates by geographic subregions. (ASIRsr), being the reference population the sum of the inhabitants under 20 years of age of Puebla, Tlaxcala, and Oaxaca in the 2020 census according to the National Institute of Statistics and Geography (INEGI). This method addressed not only the instability of standardized incidence rates in data-scarce locations but also identified the state subregions with the greatest incidence rates of AL.

**Figure 1:**
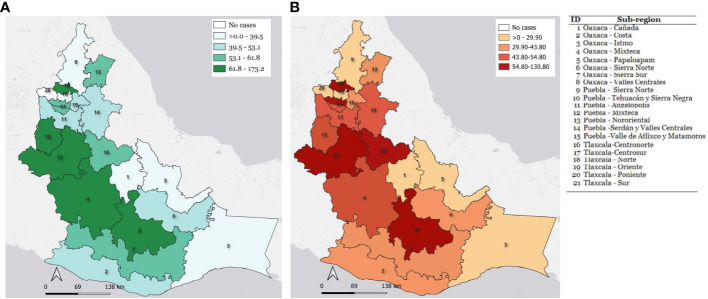
Choropleth maps representation of standardized incidence rates of childhood acute leukemia reported for each state subregion. **(A)** Incidence rates by sub-regions in children with AL from 0-14 years of age. **(B)** Incidence rates by sub-regions in children with AL from 0-19 years of age.

Finally, to know the spatial distribution of the AL incidence in each state subregion, the ASIRsr results were mapped using the QGIS 3.26 software (Open-Source Geospatial Foundation, Beaverton, Oregon, USA, QGIS, RRID : SCR_018507) (**Figure 1**).

## 3 Results

**Figure 2:**
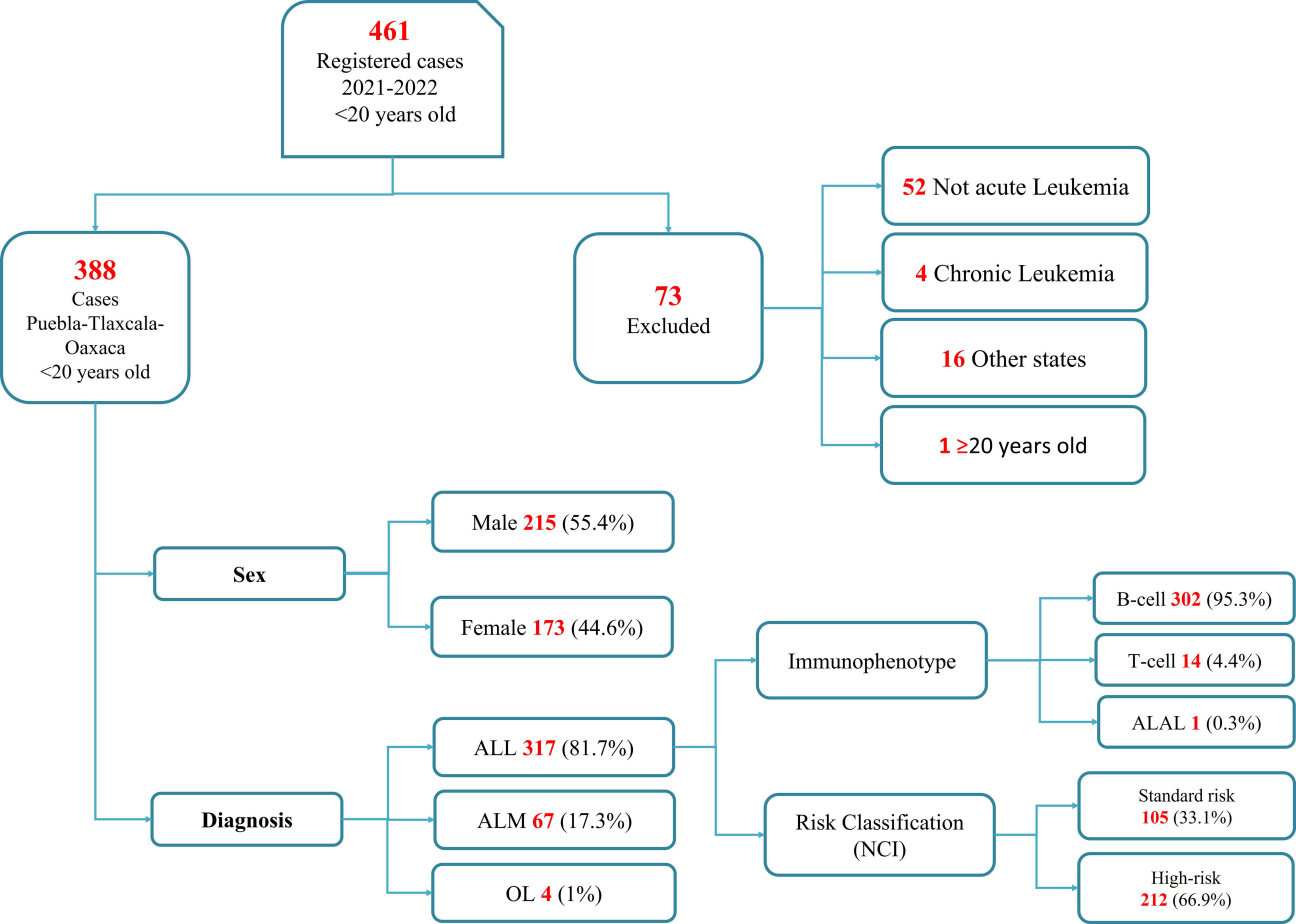
Flowchart of the selection process of children diagnosed with acute leukemia in the states of Puebla, Tlaxcala, and Oaxaca during the period 2021-2022. NCI, National Cancer Institute Classification.

During the period 2021 to 2022, 461 cases with suspected childhood AL were registered in the participant institutions, still, 73 cases were not included in the present analysis because AL was discarded, some patients were from a different state of residence or had an age greater than 20 years old at the moment of diagnosis confirmation (**Figure 2**). Consequently, a total of 388 cases had a confirmed diagnosis of AL. Among these cases, 55.4% (215) were male. The predominant AL subtype was ALL in 81.7% (n=317), followed by AML with 17.3% (n=67), and finally OL with 1% (n=4). According to the immunophenotype for the ALL subgroup, B-cell leukemias represented 95.3% (n=302), followed by T-cell with 4.4% (n=14) and ALAL with 0.3% (n=1).

### 3.1 Incidence rates by age groups

#### 3.1.1 0 to 14 years

The global AL incidence rates of the three regions in this age group were the following: a cIR of 51.4 and an ASIRw of 51.5 cases per million. Regarding the most frequent subtype of AL, it was ALL with an ASIRw of 41.5; likewise, the most frequent immunophenotype for these states was B-cell, reporting an ASIRw of 39.6. The rest of the crude and standardized rates are displayed in **Table 2**.

**Table 2:** Incidence rates of acute leukemias in children under 20 years of age from the states of Puebla, Tlaxcala and Oaxaca during the period 2021-2022.

AL	Sex	Age-specific rate								0 - 14y		ASIRw 0-14	0 - 19y		ASIRw 0 -19
		0 - 4		5 - 9		10 - 14		15 - 19							
		n	cIR	n	cIR	n	cIR	n	cIR	n	cIR		n	cIR	
All-AL	Male	52	47.77	63	56.26	71	62.39	29	25.84	185	55.40	55.28	215	48.11	48.17
	Female	57	54.26	43	39.77	53	48.01	20	18.17	153	47.29	47.39	173	39.90	40.24
	Total	109	50.96	106	106.00	124	55.31	49	22.04	338	51.35	51.46	388	44.07	44.26
Subtype															
ALL	Male	48	44.10	52	46.44	56	49.21	26	23.17	156	46.56	46.62	182	40.73	40.83
	Female	47	44.74	35	32.37	35	31.71	18	16.35	117	36.16	36.34	135	31.13	31.45
	Total	95	44.41	87	87.00	91	40.59	44	19.79	273	41.48	41.53	317	36.00	36.21
AML	Male	4	3.67	11	9.82	14	12.30	3	2.67	29	8.56	8.37	32	7.16	7.12
	Female	9	8.57	8	7.40	16	14.49	2	1.82	33	10.20	10.13	35	8.07	8.10
	Total	13	6.08	19	19.00	30	13.38	5	2.25	62	9.27	9.33	67	7.61	7.60
Other	Male	0	––––	0	–––	1	0.88	0	–––	1	0.29	0.30	1	0.22	0.22
	Female	1	0.95	0	–––	2	1.81	0	–––	3	0.93	0.92	3	0.69	0.69
	Total	1	0.47	0	–––	3	1.34	0	–––	4	0.61	0.60	4	0.45	0.45
Immunophenotype															
B-cell	Male	46	42.26	49	43.76	52	45.69	25	22.28	147	43.89	43.93	172	38.49	38.60
	Female	47	44.74	35	32.37	31	28.08	17	15.45	113	34.92	35.15	130	29.98	30.33
	Total	93	43.48	84	84.00	83	37.02	42	18.89	260	39.50	39.59	302	34.30	34.53
T-cell	Male	2	1.84	3	2.68	3	2.64	1	0.89	8	2.38	2.39	9	2.01	2.02
	Female	0	0.00	0	0.00	4	3.62	1	0.91	4	1.24	1.19	5	1.15	1.12
	Total	2	0.94	3	3.00	7	3.12	2	0.90	12	1.82	1.80	14	1.59	1.58
ALAL	Male	0	–––	0	–––	1	0.88	0	–––	1	0.30	0.29	1	0.22	0.22
	Female	0	–––	0	–––	0	–––	0	–––	0	—	–––	0	–––	–––
	Total	0	–––	0	–––	1	0.45	0	–––	1	0.15	0.15	1	0.11	0.11

AL, acute leukemia; ALL, acute lymphoblastic leukemia; AML, acute myeloid leukemia; ALAL, acute leukemias of ambiguous lineage.

#### 3.1.2 0 to 19 years

Subsequently, in this age group, the AL cIR was 44.1 cases per million for the three states and an ASIRw of 44.3. The ASIRw for ALL was 36.2, and for the B-cell subtype an ASIRw of 34.5.

### 3.2 Age incidence peaks by AL main subtypes

Two age incidence peaks for ALL were observed, the first between the ages of three and five years and the second between 13 and 16 years of age. For AML, also two incidence peaks were observed, a first at one year of age and a second at the age of 10 years.

### 3.3 Incidence rates by age group and states

#### 3.3.1 0 to 14 years

State of Puebla: The cIR for AL was 53.3 cases per million, and when the rate was standardized, it showed an ASIRw of 53.2. Regarding the AL subtype, again, it was ALL the most common subtype with an ASIRw of 45.2. The B-cell immunophenotype showed an ASIRw of 43.0 (**Supplementary Table 1**).

State of Tlaxcala: The AL cIR in Tlaxcala was 53.4 cases per million with an ASIRw of 54.7. On the other hand, the ASIRw for the ALL subtype was 41.3 and for the B-cell subtype was 39.9 (**Supplementary Table 2**).

State of Oaxaca: The observed cIR for AL was 47.5 cases per million, and when analyzing the ASIRw it was 47.7 cases per million. The ALL subtype presented an ASIRw of 35.7; also, the B-cell immunophenotype had an ASIRw of 34.0 (**Supplementary Table 3**).

#### 3.3.2 0 to 19 years

State of Puebla: This state showed a cAIR of 46.3 cases per million for AL, and when the rate was standardized, it was observed an ASIRw of 46.4. Regarding the AL subtypes, ALL presented an ASIRw of 39.6, and the B-cell immunophenotype had an ASIRw of 37.7 (**Supplementary Table 3**).

State of Tlaxcala: The AL cIR for Tlaxcala was 48.0 and an ASIRw of 48.2 per million. On the other hand, the ASIRw for the ALL subtype was 37.1, and the ASIRw for B-cell of 36.0 (**Supplementary Table 2**).

State of Oaxaca: An AL cIR of 39.3 cases per million was observed for this age group with an ASIRw of 39.6 cases per million. The ALL subtype presented an ASIRw of 30.6; also, the B-cell immunophenotype group had an ASIRw of 28.9 (**Supplementary Table 3**).

### 3.4 AL incidence rates by state subregions

The crude and standardized incidence rates of acute leukemia were reported for each subregion and represented on a choropleth map (**Figure 1**).

In the age group from 0 to 14 years, the Mixteca (Puebla) presented one of the highest rates, reporting an ASIRw of 96.7, followed by three regions with rates above 60 cases per million: the Mixteca Oaxaqueña (ASIRw 69.0), Valles Centrales of Oaxaca (ASIRw 67.0), Valle de Atlixco-Izúcar de Matamoros (ASIRw of 66.0) (**Figure 1**).

For the age group from 0 to 19 years, three regions stand out for their high rates: the Mixteca (Puebla) with an ASIRw of 88.5 cases per million, followed by the Center-South (Tlaxcala) with an ASIRw of 60.0, and ultimately, the region of Valles Centrales of Oaxaca with an ASIRw of 58.1 (**Figure 1**).

Remarkably, the North region of Tlaxcala showed an extremely higher rate in both age groups (0-14 and 0-19), with an ASIRw of 173.2 and 130.8, respectively. However, these data should be taken cautiously to avoid the fallacy of small numbers because this region has a low number of populations under 20 years of age, which makes its rates susceptible to random fluctuation.

## 4 Discussion

To our knowledge, this is the first population-based report on the incidence of childhood AL in the south-central region of Mexico. Although we only report the incidence of two years of registration (2021-2022), we consider this study to be representative of the population since it includes the participation of all hospitals attending children and adolescents with this disease. It is worth mentioning that this study continues with its active registration of incident cases.

The incidences for AL observed in this study for the three states were very relevant, as higher rates were observed in the 0 to 14 years group with 51.5 cases per million than in the 0 to 19 years group with 44.3.

The rates for the 0 to 14 years group were observed slightly above the rates reported for the African regions (0.5-33.6). (1, 22, 23), Asia (30.7-48.2) (24–31), and Central and South America (33.3-49.8) (1, 2, 32). However, these rates are intermediate in comparison to those reported for the European regions (42.1-57.1) (33–41) and Oceania (50.0-53.5) (42–44). They are lower than the rates reported for the North American regions (31.3-65.4) (1, 9, 45–53).

On the other hand, the rates reported for AL in the 0-19 years age group were similar to those reported for the Asian (42.5) (54) and Central and South American (34.8-58.5) regions (4, 32, 55). However, they were lower than those reported in the European regions (51.2-54.1). (56, 57) and North America (26.2-64.8) (58–61).

It has already been described that there are population groups that have a higher incidence of leukemia such as children of Latin American descent (Hispanics), Oceanian Americans, and Pacific Islanders, as their rates range between 54.0-65.4 cases per million for the group of 0-14 years, we consider it relevant to note that the rates reported in this study were lower despite the fact that it is a Latin American ancestry population. (1, 9, 45, 47–50, 53). Similarly, it was observed in the 0-19 age group, which, when compared to the rates of the North American population of Latin American descent, describe rates of 55.0-60.5 cases per million, which are reported to be higher than the rates described in this study (58, 59). When the incidence rates for AL reported in this study were compared with those reported recently in GMC, it was observed that, although the rates are not low, they are not as high as those reported for children in GMC (63.3) (9). Regardless, the rates obtained in the present report could be higher considering several possible factors such as lack of access to diagnosis, poverty, geographic inaccessibility, degree of marginalization of families, and ethnicity, among others. For example, the National Council for the Evaluation of Social Policy and Development (CONEVAL) estimated in 2018 that the lack of access to health services was 20.8% for the population of the state of Puebla, 16% for the population of Oaxaca and, 13.7% in Tlaxcala (62); thus, it is not uncommon to find family histories in these types of regions with long journeys for medical care. These journeys may involve several trips using different types of transportation, including extensive pedestrian travel.

The diagnosis of childhood leukemia requires specialized care, in some cases leading the parents to visit different physicians on multiple occasions because the symptoms of the child persist. All of this may result in economic expenses that low-income families often cannot afford, leaving uncertainty about the child´s diagnosis. This and other social factors become very relevant to take into account when incidence rates of leukemias are reported. Particularly, the incidence rate of AL obtained in the present study for the state of Oaxaca could be underestimated not only as a consequence of the previously mentioned factor. Oaxaca has a lot of municipalities and most of them are far from the center of the state where specialists in hemato-oncological diseases are commonly found (63). It could then be possible that children from these regions of Oaxaca become ill with leukemia and do not have the opportunity to receiving a confirmation of their diagnosis, and leukemia-associated deaths are being attributed to other diseases with similar symptomatology (64).

These situations may also occur in the states of Puebla and Tlaxcala. For example, the Mexican death system reported 1169 deaths that were certified with causes of death of nonspecific signs and symptoms (ICD10 codes, R00-R99), in the three study states, in children under 20 years of age, during the period 2015-2019 (65). This phenomenon has been reported by other investigators who have mentioned that while cancer incidence rates are higher in urban areas, mortality rates are higher in rural areas (66, 67). In addition, it has also been reported that patients in rural areas are frequently diagnosed at later stages, are less likely to receive standard treatments, optimal medical follow-up, or support services, and therefore experience worse health outcomes compared to non-rural patients (68, 69).

Another important aspect to note from the results of the present study is in relation to the biphasic pattern of age peak of incidence observed for ALL and AML. The first peak of presentation for ALL and AML are consistent with that reported in North American and European populations (between 2 to 6, and between 1 to 4 years old, respectively). Importantly, these age peaks have been related to good prognostic factors and specific cytogenic aberrations. (70–72). In ALL, the second age peak was between 9 and 14 years, while for AML it was between 7 and 13 years. These findings are consistent with what has been previously reported in the Mexican population of GMC, 11-14 years for ALL and 10-12 years for AML. (9). Remarkably, in this study, the second peaks of presentation had a longer duration (5 years for ALL and 6 years for AML) than in the study conducted in GMC (3 years for ALL and 2 years for AML) and their peak incidence points were significantly higher: at 13 years of age with a cIR 71 cases per million for ALL and at 10 years of age with a cIR 20 in AML. It is well known that the age at diagnosis of childhood AL is an important predictor of poor outcomes. Then, it would be important to identify the survival rates of adolescents in these country regions which could be lower than in other states. Additionally, it would be relevant to study the environmental factors related to the presentation of the disease during adolescence.

The regions of Puebla, Tlaxcala, and Oaxaca have a multicultural origin since their population has been made up of a mixture of different indigenous populations, as well as European and, to a lesser extent, African peoples (73, 74). Moreover, the region presents significant socioeconomic inequalities based on ethnic origin which results in high poverty rates and marginalization in different areas. Recently, according to official data from Mexico, it was reported that a high proportion of the population in the states of Puebla, Tlaxcala, and Oaxaca live in poverty (62.4%, 59.3%, and 61.7%, respectively) and these frequencies are higher than the national average (36.3%), which, among other aspects, makes these regions to be considered as marginalized populations (75–77).

Mexico does not have a census of indigenous population, the closest indicator being the proportion of households that speak indigenous languages from INEGI’s population census. With this reference, it is known that 6.1% of the Mexican population at the national level speaks an indigenous language, while in Puebla it is 9.9% with 7 native Amerindian ethnic groups, Tlaxcala 2.2% with 2 ethnic groups, and Oaxaca 31.2% with 16 ethnic groups and an important group of Afro-descendant population (4.7%) (78). From the results of the present study, the subregions with the highest incidence rates were located in the center of the study region, in the area connecting the capital cities of Puebla and Oaxaca. The Mixteca region (Puebla) showed an AL incidence rate of 96.7 and 88.5 cases per million, for groups 0-14 and 0-19, respectively. Likewise, the Northern region (Tlaxcala) reports rates of 173.2 and 130. 8 cases per million for the 0-14 and 0-19 age groups, respectively; however, these last rates should be taken with caution, because although they are high, we think that they may also be influenced by the concentration of cases due to the search for medical care between regions; or it is simply a random fluctuation due to the short time of registration along with a low population base of the Northern region (Tlaxcala) (23,000 inhabitants under 20 years of age). Nevertheless, further registration of incident cases is mandatory.

### 4.1 Study limitations and strengths

One of the possible limitations of this study could be that hospitals in nearby states were not included due to the logistics of the project; this would imply the loss of some cases of resident children who migrated to neighboring states in search of medical attention. However, the observed rates were high despite this limitation, in addition to the fact that we counted the participation of all public and private hospitals in the region that attend to patients with this condition. Additionally, we also have the participation of public hospitals located in GMC. GMC represents the center of the country and is close to the states of Puebla and Tlaxcala. These hospitals have specialists in pediatric hematology and oncology as well as the infrastructure for the diagnosis and treatment of children from these and other states of the country.

The inclusion of death certificates as a supplementary source of information for the registry was unfeasible given the fact that in Mexico the information from death certificates becomes available at least two years after the conclusion of the relevant year of interest. Notwithstanding, death certificate information serves as an additional avenue for identifying incident cases of neoplasms that may be diagnosable in alternative health institutions or laboratories not encompassed within our study. Furthermore, acute leukemias represent pathological entities exclusively diagnosed within highly specialized hospitals or laboratories, akin to the participating institutions in the present research. On the other hand, the methodology employed in the present study entailed an active process carried out by trained personnel consisting of cross-referencing daily hospital admission registries and patient lists within each pediatric hematology and oncology department. This is to ensure the identification and follow-up of all the potential cases with acute leukemia until diagnosis confirmation.

Notably, we consider that a strength of this study was to follow quality control recommendations for the validation of population-based cancer registries: a) comparability, b) internal consistency, c) validity, and d) timeliness (16, 17). In the first instance, to maintain comparability among studies international standard classification and coding systems of neoplasms (ICD-O3 and ICCC-3) were considered in the present research. Additionally, according to the agreed standards about the definition of the incidence date, in the present study, the date of leukemia confirmation through the morphological study of the bone marrow aspirate was used. Secondly, internal consistency was checked. For this purpose, the registrars were standardized before the study initiation, and they were supervised by a local coordinator on a weekly basis by corroborating the information through phone calls, clinical charts, and laboratory data before they were reported in the central database. Likewise, the validity of the information was gained through the active and systematic revision of the morphological study of the bone marrow aspirates, histochemistry staining, and the immunophenotype which are routinely performed in the participant institutions. The percentage of cases with a morphologically verified diagnosis was 100%. Furthermore, the rate of cases with missing data was 0%. In addition, we collected information regarding the cause of death within the study population. Finally, regarding timeliness, in the present investigation rapid reporting was achieved considering that the average time between case confirmation and report in the central database was seven days. Consequently, the data obtained in this study has been collected with sufficient rigor to constitute a first reference of the descriptive epidemiology of childhood acute leukemias in the states of Puebla, Tlaxcala, and Oaxaca.

## 5 Conclusion

The present work is a robust source of information on the incidence of acute leukemia in the south-central region of the Mexican Republic. These rates are within the range reported in other populations of Latin American origin. Two age peaks of occurrence were observed in the first years of life and a second peak after 7 years of life. Additionally, differences were observed between subregions that could be attributed to social factors linked to the ethnic origin of the populations, but further research is mandatory to elucidate this hypothesis. Finally, we consider this a valuable report for the planning of care and research strategies for acute leukemias in the region.

## Data availability statement

The raw data supporting the conclusions of this article will be made available by the authors, without undue reservation.

## Ethics statement

The studies involving humans were approved by National Scientific Research and Ethics Committee of the Mexican Institute of Social Security. The studies were conducted in accordance with the local legislation and institutional requirements. Written informed consent for participation in this study was provided by the participants’ legal guardians/next of kin.

## Author contributions

JF-L: Conceptualization, Data curation, Formal analysis, Investigation, Methodology, Supervision, Writing – original draft, Writing – review & editing. AA-L: Conceptualization, Data curation, Formal analysis, Investigation, Supervision, Writing – original draft, Writing – review & editing, Methodology. DD-R: Writing – original draft, Writing – review & editing, Formal analysis, Investigation, Methodology. EA-Ru: Writing – original draft, Writing – review & editing, Formal analysis, Investigation, Methodology. LL-C: Writing – review & editing, Investigation. TS-L: Investigation, Writing – review & editing. MC: Investigation, Writing – review & editing. MB-L: Investigation, Writing – review & editing. DC-A: Investigation, Writing – review & editing. JE-R: Investigation, Writing – review & editing. JL-A: Investigation, Writing – review & editing. MG-H: Investigation, Writing – review & editing. DO-C: Investigation, Writing – review & editing. VT-C: Investigation, Writing – review & editing. KM-J: Investigation, Writing – review & editing. PA-C: Investigation, Writing – review & editing. EA-Ro: Investigation, Writing – review & editing. WH-O: Investigation, Writing – review & editing. GR-A: Investigation, Writing – review & editing. LC-A: Investigation, Writing – review & editing. AM-T: Investigation, Writing – review & editing. LC-C: Investigation, Writing – review & editing. NL-S: Investigation, Writing – original draft. MM-M: Investigation, Writing – review & editing. AR-R: Investigation, Writing – review & editing. LM-P: Investigation, Writing – review & editing. CG-D: Investigation, Writing – review & editing. AM-S: Investigation, Writing – review & editing. MG-R: Investigation, Writing – review & editing. JM-T: Investigation, Writing – review & editing. ER-C: Investigation, Writing – review & editing. VB-M: Investigation, Writing – review & editing. MR-T: Investigation, Writing – review & editing. AC-M: Investigation, Writing – review & editing. GJ-A: Investigation, Writing – review & editing. SP-T: Funding acquisition, Investigation, Resources, Supervision, Writing – review & editing. JR-E: Investigation, Writing – review & editing. MS-A: Investigation, Writing – review & editing. FH-Q: Investigation, Writing – review & editing. AH-D: Investigation, Writing – review & editing. LG-G: Investigation, Writing – review & editing. MM-R: Investigation, Writing – review & editing. AO-S: Investigation, Writing – review & editing. HR-V: Investigation, Writing – review & editing. SJ-M: Investigation, Writing – review & editing. MC-G: Investigation, Writing – review & editing. MÁ-B: Investigation, Writing – review & editing. CD-M: Investigation, Writing – review & editing. RP: Funding acquisition, Investigation, Resources, Supervision, Writing – review & editing. JM-A: Conceptualization, Data curation, Formal analysis, Funding acquisition, Investigation, Methodology, Project administration, Resources, Supervision, Validation, Visualization, Writing – original draft, Writing – review & editing. JN-E: Conceptualization, Data curation, Formal analysis, Funding acquisition, Investigation, Methodology, Project administration, Resources, Supervision, Validation, Visualization, Writing – original draft, Writing – review & editing.
